# Recherches Anatomiques sur l'Emphyseme Pulmonaire

**Published:** 1839-04-01

**Authors:** 


### Recherches Anatomiques sur l'Emphyseme Pulmonaire. Par le Dr. *Lombard,* Medecin de l'Hôpital Civil et Militaire de Geneve. 4to. Geneva, 1838, with Plates.

### Anatomical Researches on Pulmonary Emphysema.

The character of Dr. Lombard, as a zealous and successful cultivator of thoracic pathology, more especially as the author of some curious researches on the nature of tubercular phthisis, is so well known, that we think it unnecessary to offer any apology for the analysis we are now about to present of a very interesting essay by that gentleman on pulmonary emphysema. The symptoms, causes, and every thing regarding the nosological history of this affection having been very recently detailed in the writings of Dr. Stokes and M. Louis, our author disclaims all intention of attempting any improvement on the opinions advanced by these writers. He professes to confine himself strictly to the anatomical history of the affection. In 1821 Magendie, in the Journal de Physiologie, T. 1, showed that the number and size of the pulmonary areolæ varied considerably with the age of the individual, so that the lung which is very dense in the infant, becomes less so in the adult, and even rarified in the aged. A memoir by MM. Hourmann and De Chambre in the Archives de Med. 1836, have further confirmed M. Magendie's views with respect to the atrophy of the lung in old persons. Dr. Lombard conceives that pulmonary emphysema is a phenomenon closely allied to this state of atrophy of the lung; only it attacks children and adults as well as, though more rarely than, old persons; and though the lungs of the latter seldom present so great a dilatation as that of emphysematous lungs, still he thinks the nature of the anatomical lesion of the pulmonary tissue the same in both. Notwithstanding the author's very modest statement that he intends to confine himself strictly to the anatomical history of this affection, he has proposed some very ingenious views accounting for the symptoms that accompany it, and the treatment he would recommend for its prevention and relief.

Before commencing his subject the author apprizes us, that under the head of *pulmonary emphysema,* he does not mean to include that which has been styled by Laennec *interlobular emphysema,* which, as it exists outside the pulmonary tissue, he conceives should not be set down as a lesion of the organ of respiration. He divides his essay into *four* sections—in the first he gives the "Anatomical Details regarding Pulmonary Emphysema."—2nd. "The Theory of the Formation of Pulmonary Emphysema."—3rd. "Explanation of the Symptoms of Pulmonary Emphysema by the nature of the Anatomical Lesion."—4th. "Practical Inferences with respect to the Treatment of Pulmonary Emphysema."

1. With respect to the *anatomical details* the author observes that, pulmonary emphysema presents itself under three very distinct forms, according to the extent of the lung affected; when the lesion is confined to some isolated vesicles, the emphysema may be called *vesicular;* when an entire lobule is affected, and this is the most frequent case, we have *lobular* emphysema; and when an entire lobe is the seat of this lesion, he distinguishes it by the name of *lobar* emphysema. These three designations he prefers to those of general and partial emphysema as hitherto employed.

1. *Vesicular Emphysema.*—The pulmonary vesicles, when they have become emphysematous, are sometimes isolated, sometimes collected together in groups of three or four; their seat is most usually the thin edge of the lung; they are found, however, in all the parts of a lobe: situated on the internal surface of the lung, they raise the pleura, and form there small bladders somewhat resembling those of *Pemphigus.* These vesicles are really larger than they appear to be, as the portion of the vesicle contained in the pulmonary tissue is usually greater than that portion which projects externally. When these vesicles come to acquire considerable size, they result uniformly from the union of several cells, the intermediate septa of which have been broken down, and they then come under the head of our author's second division, that of emphysema occupying an entire pulmonary lobule. The cases of isolated dilatation of one vesicle, and which might result from real hypertrophy, our author thinks, must be very rare, as he has never met with a single instance of it, and as in all those cases where he has had an opportunity of dissecting a vesicle apparently simple, he always found it multiple, its internal surface presenting anfractuosities, which he found to be the vestiges of former intervesicular septa.

2. *Lobular Emphysema.*—This form is much the most frequent, it consists in the development of all the vesicles of a pulmonary lobule. By this it is not meant that all the air-cells are in the same degree of development, but only that the entire lobule participates more or less in the morbid state. The author gives figures illustrating this form of lesion, and likewise exhibiting the independence of each lobule with respect to the development of the emphysema.

The great development of an emphysematous lobule accounts for the formation of the vesicular appendices found attached to the thin edges of the lungs. The hydatiform appendices vary considerably in size, some being only a few lines in diameter, whilst others of them are several inches in circumference. By attentively examining the pedicle which unites these appendices to the lung, it will be found in some cases formed by a dense cellular tissue, containing no trace whatever of air vesicles; in other cases it will be possible to recognise in the pedicle air vesicles which are not obliterated, so that there exists a communication between the emphysematous appendix and the rest of the lung. This pedicle is sometimes a few lines in length, at other times it is so short, that the vesicle seems to be attached immediately to the lung. The appendix itself is formed by a simple or multiple cavity; the latter case is by much the most frequent. In the hydatiform appendices, he tells us, all the varieties of emphysema are found, from the unequal dilatation of some cells to the destruction and fusion of all the vesicles into one, the parietes of which are anfractuous and traversed by thin transparent filaments.

The cavities of the emphysematous lobules present all the degrees of development, from the fractions of a line to one or two inches in diameter. When carefully examined, these cavities, far from being simple and regular, are always multiple and anfractuous; they often acquire also considerable size. These different appearances the author illustrates by figures.

When in an emphysematous lobule there is but a certain number of cells increased in size, it is, as our author observes, the most superficial that are so, and in general the lesion is so much the more marked, as we pass from the centre to the circumference. Our author does not recollect ever having met with a lobule emphysematous in the centre, and normal at the edges of the lung. Why the development of the most superficial vesicles is easier and greater than that of the central cells, he accounts for in this way: because at the surface of the lung there is only the pleura to oppose the extension of the pulmonary tissue, which, being a serous membrane, is very extensible, whilst such is not the case with the tissues situate at the centre of the lung.

3. *Lobar Emphysema.*—When the Emphysema extends to an entire lobe, it presents two very distinct varieties: in the first, all the lobules are emphysematous, but in very different degrees; this is the most ordinary case. The second form of lobar emphysema is that which has transformed the entire tissue of a lobe or even of a lung into a spongy body, so large that one might think it to be hypertrophy; but the increase in size depends not on an addition to, but rather on atrophy of, the areolar tissue, which, having lost its elasticity, allows itself to be distended by the inspired air. This form of lobar emphysema is that which approaches nearest to the normal state of the lung of old persons.

With respect to the first variety, which is the product of the union of several emphysematous lobules, the description already given of lobular emphysema will apply. In regard to the uniform dilatation of an entire lobe, our author states that this form presents different circumstances which it is important to notice. In the first place the vesicles, though apparently uniform in figure and size, are far from having all of them the same dimensions; some are three or four times their normal size, whilst others are scarcely at all augmented in size; in certain portions of the lung distinct anfractuosities are found, whilst in other parts the tissues present no very apparent solution of continuity. A second circumstance observed in lobar emphysema is the obliteration of the blood-vessels, or at least their diminution in number and in size: the pulmonary tissue in the normal state is of a deep red colour, and traversed in every direction by numerous capillaries, whilst in the emphysematous state we have an areolar tissue, white and almost completely bloodless. Again, a third character of this form of lobar emphysema consists in the destruction, or at least in the fusion, of the cellular tissue which separates the lobules in the normal state, which makes the lung appear uniform and without intersection through the entire extent of a lobe.

### II. Theory of the Formation of Pulmonary Emphysema.

Our author, after describing the different forms of pulmonary emphysema, next proceeds to consider this disease in itself, and to investigate the state of the pulmonary tissue which constitutes emphysema. The great development of the air vesicles must be owing either to hypertrophy, or to the union of several cells isolated in the normal state, but united into one cavity by the destruction of the intermediate parietes. Laennec and Andral recognise both these origins of pulmonary emphysema, whilst M. Louis considers hypertrophy as the most ordinary state of emphysematous lung.

"If," says our author, "we examine with the microscope a piece of emphysematous lung previously dried, we shall find, at first sight, that the increase in the size of the lung is not owing to the thickening of the tissue of this organ, and that far from finding a dense and resisting tissue, as it should be if it were hypertrophied, we find, on the contrary, a porous, light tissue, the intersections of which are either destroyed or so attenuated as to become transparent."

The attenuated and transparent state of the intervesicular parietes of an emphysematous lung would give rise to the idea of a very extensive obliteration in the blood-vessels; but theory could reveal only a part of the modifications which the pulmonary tissue has undergone in this respect. Observation shews that obliteration of almost all the blood-vessels is the essential character of pulmonary emphysema. The tissues lose their thickness, their colour, and their elasticity, they become thin and transparent in consequence of the destruction of the blood-vessels, which, in the healthy state, ran through them in all directions, and formed of them a real erectile tissue.

Thus direct observation leads our author to recognise in pulmonary emphysema two anatomical circumstances essential to its existence: 1st, the destruction of a considerable part of the intervesicular parietes, and the union into one anfractuous cavity of a greater or less number of air-cells originally separated; 2nd, the obliteration of almost all the capillaries in the emphysematous portions of the lung. So that in order to trace the anatomical history of pulmonary emphysema all that now remains is to discover the relations which connect these two facts, and more especially to investigate whether they are to be considered in the light of cause and effect, by ascertaining whether one of them has necessarily preceded the other.

In investigating these points our author observes that, if destruction of the intervesicular parietes had preceded the obliteration of the blood-vessels, hæmoptysis should be one of the most constant symptoms of the commencement of pulmonary emphysema; and yet out of thirty-five cases under M. Louis' care, only one had hæmoptysis, and this individual at a subsequent period presented evident symptoms of tubercles; from which it may be inferred that hæmoptysis is not one of the symptoms of emphysema, as should be the case if obliteration of the blood-vessels had not preceded the destruction of the tissues traversed by them. "And let it not be said," says our author, "that in emphysema the portion of the pulmonary tissue destroyed contains only blood-vessels too small to give rise to any perceptible hæmorrhage, as we see entire lobules transformed into a pouch of three or four inches in circumference, the cavity of which is large enough to contain a nut and always remains open." Thus then obliteration of the blood-vessels must be considered as the first degree of the formation of pulmonary emphysema. The author now endeavours to show how the destruction of the intervesicular parietes is the natural consequence of this occurrence. It is of constant observation in pathology that when an organ is rendered useless, it becomes atrophied and ultimately disappears. This is what happens to the pulmonary tissue when it is no longer traversed by the capillary bloodvessels, the parietes of the vesicles become attenuated and at length disappear, the result of which is the formation of the anfractuous cavities which constitute pulmonary emphysema. These attenuated and atrophied membranes are then observed to lose their elasticity, and become incapable of expelling the air which remains in them during expiration; thence arises a new series of phenomena which has led some anatomists to consider emphysema as an atrophy of the pulmonary tissue.

The air which penetrates a healthy lung is driven out during each expiration by virtue of the elasticity of the pulmonary tissue. In an emphysematous lung the air enters irregular, anfractuous cavities, the parietes of which have no longer strength to expel it; hence arise two important phenomena: the first is the state of permanent tumefaction of an emphysematous lung; the weight of the atmosphere, which, in the healthy state, when the sternum is raised, is sufficient to press down the lung, is insufficient to expel the air imprisoned in a lobule or an emphysematous lobe; thence comes an increase of size which is more apparent than real, and which gives to the lung thus changed the appearance of a tissue inflated with some force, and which occupies a much greater extent than in the normal state. The air which fills the air vesicles renders it more difficult to tear, for the same reason that a healthy and crepitating lung is more resistant under pressure than an engorged or hepatised lung. There is then in an emphysematous lung an apparent increase of size, but which no more depends on hypertrophy of tissue than the temporary erection of the nipple or of the penis.

In the next place, besides the phenomenon just mentioned, and the object of which is principally lobar emphysema, there is another observed in the emphysematous lobules, when the latter, instead of retaining their primary form, protrude through the lung, and really increase in size; but there is not, in this case, a real hypertrophy, since the pulmonary tissue so far from being more dense or solid, is, on the contrary, distended beyond measure, and almost completely destroyed. This increase of size is probably owing to the expansion of the air imprisoned in a tissue whose temperature is much higher than that of the atmosphere; but further, the normal development of an emphysematous lobule is particularly favoured by the diminished elasticity of the tissues, which are no longer traversed in all directions by numerous venous and arterial capillaries. The efforts of coughing might be supposed to perform an important part in this phenomenon; but the history of the symptoms contradicts this opinion, since in one fourth of the patients observed by M. Louis, the cough which might have occasioned the destruction of the air-cells, did not commence till after the dyspnœa, and when the emphysema might be considered as already formed.

### III. Explanation of the Symptoms of Pulmonary Emphysema by the Nature of the Anatomical Lesion.

If the different facts contained in these anatomical researches be now recapitulated, it will be seen that pulmonary emphysema is a morbid state of the lung which commences by the obliteration of the capillary blood-vessels, and which, at a later period, destroys the air-cells, and changes them into vast membranous and irregular cavities, so that one is led to consider pulmonary emphysema as *a partial destruction of the organ of respiration*, a destruction which renders the lung completely useless for the functions of hæmatosis. Pulmonary emphysema, far from being an hypertrophy, is then a real atrophy, and this opinion is so well founded on facts, that in an emphysematous lung, we find, beside the tumefied lobules, the vestiges of other lobules, which have completely disappeared from atrophy of the tissues. Such is the opinion which our author has arrived at by direct observation of emphysematous lungs: he next endeavours to trace the relations which connect pulmonary emphysema with the symptoms observed in persons whose lungs have undergone this degeneration.

The symptoms of emphysema may be divided into two classes: the first class comprising those which are the immediate consequence of the prolonged sojourn of air in the lung; the others, though connected probably with this circumstance, are not the direct result of insufficiency of respiration.

With the first class are connected sonorousness of the chest, the absence of the respiratory murmur, the abnormal development of the thoracic parietes, and debility of the muscles of inspiration noticed by Dr. Stokes as the consequence of the forced extension in which they are kept by the morbid development of the lungs.

In the second class may be reckoned dyspnœa, frequency of pulmonary catarrhs and cough, which is the consequence of the latter, palpitations, hypertrophy of the heart, and œdema of the lower extremities.

On comparing these two classes of symptoms with the anatomical state of the lung as already described in this article, it will be found how easy it is to account for the production of the different symptoms now enumerated. It has been shown that emphysematous lungs are transformed into irregular cavities circumscribed by a thin, membranous tissue, which has lost the elasticity necessary to drive out the air conveyed to them by inspiration; besides, this air being heated by its sojourn in a body whose temperature is very high, acquires a much greater size. From these two physical facts result all the symptoms of the first class: the sonorousness of the chest, a considerable quantity of air remaining confined in the lung; the abnormal development of the chest, it being a matter of constant observation that the parietes of the cavities are moulded on their contents, and that if the lung keeps itself permanently in a state of forced distention, the corresponding portion of the chest must become developed in the same proportion. The absence of the respiratory murmur is the natural consequence of the fulness of the lung, which being already distended beyond measure by the atmospheric air, can no longer receive any at each inspiration. The weakness of the inspiratory murmur appears also to be connected with debility of the inspiratory muscles. There is not then any one of the symptoms of the first class which is not satisfactorily explained by the morbid state of the emphysematous lung. Those of the second class are also an evident consequence of it, though less directly than the preceding; for they result, some from the obstruction occasioned to the circulation by the obliteration of the greater part of the capillaries of the lung, and by the forced distention of the lung; this will account for the palpitations, hypertrophy of the heart, and the anasarca which is the consequence of it; the others arise from the obstacle caused to the respiration by the state of permanent development in which the lung is kept in emphysema; in this way our author accounts for the dyspnœa, cough, and pulmonary catarrhs so frequent in emphysematous patients; the latter symptoms may also, he conceives, be the result of the increased activity of the healthy parts of the lung in persons in whom an entire lobe, and oftentimes an entire lung, has become completely useless for the function of hæmatosis.

### IV. Practical Consequences respecting the Treatment of Pulmonary Emphysema.

Our author now applies the preceding remarks to the treatment of pulmonary emphysema. Those who consider this disease hypertrophy by passive dilatation of the vesicles, endeavour to restore tone to the bronchial muscles and for this purpose they recommend strychnine; this medicine however can evidently be of no use to re-establish the septa of the vesicles which have disappeared; probably however it might be employed to restore a little tone to the thoracic muscles from its marked and striking influence on the spinal cord, and consequently on the nerves which it sends to the respiratory muscles. The author never tried this mode of treating the disease. Louis employs opium, which possesses decidedly beneficial properties with respect to diminishing the difficulty of breathing in emphysematous patients. Dr. Lombard supposes that it may produce this effect by diminishing the physical want of respiration, and consequently by putting a stop to that state of spasm in which the anxiety of the patient keeps all the respiratory muscles. Laennec used to recommend polygala, oxymel of squill, &c. in order to diminish the dyspnœa in emphysematous subjects: the only effects however such medicines could have is to diminish the viscidity of the sputa; it is obvious that none of these means could exercise any influence on the lesion which constitutes pulmonary emphysema.

A rational treatment, according to our author, of this disease, should, in the first place, modify the pulmonary circulation so as to prevent the obliteration of the capillary blood-vessels, and, in the next place, render the expiration more complete. Of these two indications the first is much the most difficult to be fulfilled, inasmuch as it is evident that the phenomenon it would combat is enveloped in the utmost obscurity; however, observes our author, going on the fact that obliteration of the capillaries is the natural consequence of their diminished activity, it is readily understood that if it was possible to foresee the formation of pulmonary emphysema, it would be necessary to combat this disposition of the blood-vessels to become obliterated by all those means which should increase the movement of the blood through the lung; a sharp and bracing air should be recommended to the patient; exercise continued for a long time, and so conducted as to render the respiration more complete; in a word, the tonic and strengthening treatment would be the best adapted for the first period of emphysema, and might be successfully employed in young persons whom either an hereditary predisposition or attacks of dyspnœa seemed to threaten with this disease.

The second indication, that which consists in driving out the air confined in the lung, might be fulfilled by all those means capable of rendering the respiration more complete, and above all the expiration more easy. Such are gymnastic exercises, riding on horseback, and all those exercises which tend to strengthen the muscles of the trunk. Strengthening douches on the thoracic parietes, sea bathing and stimulating frictions on the chest, might be employed in the same cases. Probably also the strychnine recommended by others for a very different reason, however, might fulfil the indication now in question by rendering the contractions of the diaphragm and of the intercostal muscles more complete and more energetic. Pulmonary catarrh and hypertrophy of the heart, when consequences of emphysema, are to be treated in precisely the same way as in one who is not emphysematous.

In conclusion, the author acknowledges that, with respect to the tonic treatment here recommended and to which he was led by the theoretical views which he has given of this affection, it has not yet received the sanction of experience, without which no method of treatment can be recommended with anything like confidence, practical medicine being one of those objects in the circle of the sciences to which unfortunately *à priori* reasoning is but little applicable.

